# HPV Vaccination Coverage in Brazil’s State of Paraná: Spatial Distribution and Advances in Public Health

**DOI:** 10.3390/vaccines12101118

**Published:** 2024-09-29

**Authors:** Fernando Castilho Pelloso, Daiane Cristina Pazin, Lincoln Luís Silva, Maria Dalva de Barros Carvalho, Deise Helena Pelloso Borghesan, Marcia Edilaine Lopes Consolaro, Lander dos Santos, Helena Fiats Ribeiro, Kely Paviani Stevanato, Vlaudimir Dias Marques, Camila Wohlenberg Camparoto, Constanza Pujals, Raissa Bocchi Pedroso, Sandra Marisa Pelloso

**Affiliations:** 1 Municipal Health Department, Curitiba 80060-130, PR, Brazil; fercaspell@gmail.com; 2 School of Medicine, Pontifícia Universidade Católica do Paraná, Curitiba 80215-901, PR, Brazil; daiane.pazin@pucpr.br; 3 Health Sciences Center, State University of Maringá-UEM, Maringá 87020-900, PR, Brazil; lincoln.luis@grupointegrado.br (L.L.S.); mdbcarvalho@gmail.com (M.D.d.B.C.); lander.santos@docentes.unicesumar.edu.br (L.d.S.); kelystevanato@gmail.com (K.P.S.); vlaudimirdm@gmail.com (V.D.M.); camila.wsouza1@gmail.com (C.W.C.); constanza.pujals@gmail.com (C.P.); raissap@gmail.com (R.B.P.); smpelloso@uem.br (S.M.P.); 4 Catholic College of Mato Grosso, Várzea Grande 78070-200, MT, Brazil; coord.enf@unifacc.com.br; 5 Department of Biomedicine, State University of Maringá-UEM, Maringá 87020-900, PR, Brazil; melconsolaro@uem.br

**Keywords:** cervical cancer, immunization, HPV

## Abstract

**Background/Objectives:** To analyze the spatial distribution of HPV vaccination coverage in relation to sociodemographic variables in a state of Southern Brazil. **Methods:** This was an ecological, retrospective study with secondary data from the Department of Information Technology of the Unified Health System/Ministry of Health from 2015 to 2022. The cohort method was used to calculate vaccination coverage. Geographically weighted regression was used for the independent variables. **Results:** There was a 22.04% reduction in vaccination between the first and second doses. Coverage with the first dose of the vaccine reached 95.17% for the female population, 64.67% for the male population, and 79.57% for both sexes. In 50.62% of cities, coverage exceeded 90% for both sexes. In 80.45% of cities, the recommended coverage for females was achieved. The variable municipal performance was positively significant for the increase in vaccination coverage in 45.45% of the regions for girls, 18.18% for boys, and 36.36% for both sexes. The family health strategy variable was significant in 9.09% of the regions for girls and both sexes. The education variable showed an inverse significance for girls in 40.90%, for boys in 18.18%, and for both sexes in 36.36% of the regions. **Conclusions:** HPV vaccination declined between the first and second doses, with high first-dose coverage among females and moderate coverage among males. Municipal performance notably impacted coverage, particularly for girls. The family health strategy was relevant in specific regions, while educational factors had a variable effect. Addressing these variables may enhance vaccination coverage and minimize the gap between doses.

## 1. Introduction

Cervical cancer ranks as the fourth-most prevalent cancer among women worldwide, with approximately 660,000 new cases and around 350,000 deaths reported in 2022 [1]. Each year in the United States, about 11,500 new cases of cervical cancer are diagnosed, and about 4000 women die of this cancer [2], while there are around 3300 new cervical cancer cases in the UK every year [3]. It is the most common cancer in women in 25 countries, many of which are in sub-Saharan Africa, and it is responsible for 51,500 deaths in the Americas [4]. Brazil has an estimated 17,000 new cases and an estimated risk of 15.38 cases per 100,000 women. However, there is some variation in the magnitude and types of cancer between regions of the world and Brazil [5]. Both mortality and incidence are related to inequalities of income, gender, access to health services, and preventive measures. Cervical cancer is associated with viral infection and can affect both men and women at some point in their lives [6].

Each year in the United States, 34,800 women and men are diagnosed with cancer caused by human papillomavirus (HPV) infection [7]. HPV causes almost all types of cervical cancer. Although most HPV infections are asymptomatic and resolve themselves within 1 to 2 years, infection with oncogenic types can lead to cancer. Cervical cancer is the only HPV-associated cancer for which screening is routinely recommended [8]. Other strategies are being adopted to eradicate cervical cancer, in which 90% of girls by age 15 are vaccinated, 70% of women aged 35 to 45 have undergone high-performance screening tests, and 90% of women with pre-cancer and cancer receive treatment [9,10].

In the last two years, actions such as immunization and cervical cancer-preventive programs have suffered negative influences, making it impossible to maintain quality parameters, care, and epidemiological records [11,12]. In this sense, the fundamental point that brings a relevant contribution to cervical cancer-preventive actions is the vaccination of priority groups. Brazil’s public immunization policies have been considered a global reference for decades and represent a significant advance in public health [13].

A study of 31,940 people showed the importance of the vaccine as a preventive measure against HPV infections, but reported that its use remains low in many countries. According to that study, the use of simpler vaccination schedules could increase the number of people vaccinated [14]. Other studies conducted in England, Scotland, and Sweden showed that the adoption of HPV vaccines in immunization program for girls aged 12 and 13 years old resulted in a considerable reduction in cervical cancer [15,16,17].

Despite the effectiveness of the vaccine and vaccination campaigns to prevent HPV following WHO goals and recommendations, countries like Brazil [18] are far from achieving the ideal 90% coverage rate intended to protect girls aged 9 to 14 years against HPV [19], especially in the state of Paraná, which has faced greater challenges in achieving vaccination coverage compared to other states [20].

Continuous surveillance of cancer associated with HPV infections can reflect on HPV vaccination coverage at state, national, and global levels, strengthen screening, and keep abreast of its long-term impact [8]. To our knowledge, no studies have discussed HPV vaccination coverage related to sociodemographic variables and their spatial analysis. Therefore, this study aimed to analyze HPV vaccination coverage in relation to sex, age group, municipal performance, educational performance, family health strategy, and primary care, with a spatial analysis in a state of Southern Brazil.

## 2. Materials and Methods

### 2.1. Study Design

This was an ecological, cross-sectional, retrospective study with spatial analysis, using secondary data on HPV immunization in Paraná. The study period was from 2015 to 2022, involving both sexes in the age group of 9 to 14 years. The research followed the guidelines of the Strengthening the Reporting of Observational Studies in Epidemiology (STROBE) protocol [21].

### 2.2. Study Population

**Figure 1:**
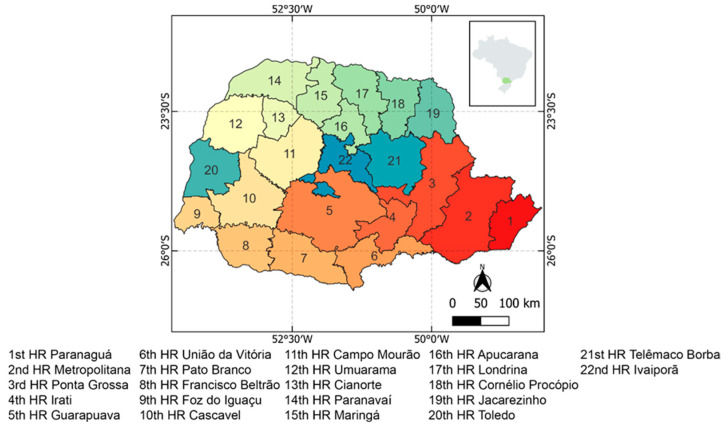
Location of the state of Paraná. It is located in the southern region of Brazil and divided into 22 health region.

The study included adolescents aged 9 to 14 years who were immunized with the quadrivalent HPV vaccine in the state of Paraná between 2015 and 2022. The state of Paraná has a total population of 11,444,380 distributed over a territorial area of 199,298.981 km^2^, according to the Brazilian Institute of Geography and Research (IBGE) [22]. It is one of the states in Brazil with the best Human Development Index (HDI) (0.749) (IPARDES, 2019). It is divided into 22 health region grouped by municipalities to facilitate the organization of health services offered to the population [23] (Figure 1).

According to estimates from the Department of Information Technology of the Unified Health System (DATASUS), in 2021, there were around 896,563 adolescents between 9 and 14 years old, of which 437,973 were female and 458,590 were male [24].

### 2.3. Study Variables

The dependent variable of this study was HPV vaccination coverage. Until 2023, the HPV vaccination schedule consisted of two doses with a minimum interval of six months. However, in 2024, the National Immunization Program (PNI), through NT 41/2024, recommended a single dose for boys and girls aged 9 to 14 and three doses for immunocompromised individuals and victims of sexual violence. For this study, we based our calculations on the previous guidelines, as vaccination data for the years 2023 and 2024 were not yet available [25].

To obtain this variable, it was necessary to perform a cohort calculation recommended by the National Immunization Program (PNI) of Brazil and the Pan American Health Organization (PAHO) [20,26]. The vaccine cohort method was used to calculate vaccination coverage (VC) of adolescents throughout the country. This method consists of accumulating vaccination data from the introduction of the vaccine until the last year evaluated. For example, in the year 2022, the 9-year-old population corresponded to the population vaccinated at age 9 in 2022. The 10-year-old population consisted of the sum of the population vaccinated at age 9 in 2021 and the population vaccinated at age 10 in 2022. Similarly, the 11-year-old population was calculated by adding the populations vaccinated at age 9 in 2020, at age 10 in 2021, and at age 11 in 2022. Finally, the 12-year-old population was determined by adding the populations vaccinated at age 9 in 2019, at age 10 in 2020, at age 11 in 2021, and at age 12 in 2022.

To explain vaccination coverage, independent variables obtained from a report of the Paraná Institute for Economic and Social Development (IPARDES) were selected. Among the IPARDES variables, data for municipal performance, education performance, primary care coverage, and coverage provided by the family health strategy were collected. The municipal performance variable is an index that evaluates three main areas of economic and social development with equal weight: education, health and income, employment, and agricultural production. The index ranges from 0 to 1, and the closer to 1, the better the municipality’s performance. The databases used are official public statistics, mainly composed of mandatory administrative records, with annual periodicity and municipal scope. The indicators were selected for their relevance to local development and their sensitivity to the actions of public agents, although the results depend on joint efforts of governments and the private sector [27].

The variable performance in education includes indicators of early childhood, primary, and secondary education, with data provided by the Ministry of Education. The population coverage estimated by the family health strategy teams is given by the percentage of the population covered by these teams in relation to the total population estimate. Finally, the population coverage estimated in primary care (PC) was given by the percentage of the population covered by family health strategy (FHS) teams and by traditional primary care teams equivalent and parameterized in relation to the total population estimate [28]. Table 1 presents the data source for the variables used in this study.

### 2.4. Data Analysis

The data were put into a spreadsheet, and subsequently descriptive statistics were applied using absolute and relative frequencies to analyze vaccination between 2015 and 2022. It was verified which municipalities achieved the vaccination coverage rate considering that the target for achieving vaccination coverage is 90% by 2030, as determined by the World Health Organization (WHO) [29].

Next, vaccination coverage was calculated and the Moran index was applied to identify whether there was spatial dependence in relation to vaccination coverage by municipalities in Paraná. This index varies between −1 and +1, and the more extreme the value, the greater the indication of spatial dependence, while values close to 0 (zero) indicate that the data are randomly distributed, with no spatial dependence [30,31]

After confirming spatial dependence, geographically weighted regression (GWR) was used to examine whether the independent variables could explain the vaccination coverage values of the municipalities of Paraná. GWR is a spatial analysis technique that allows the coefficients of the independent variables to vary across geographic space. Unlike global regression models, which assume uniform relationships across a study area, GWR recognizes that these relationships can vary spatially. This makes GWR particularly useful for understanding local variations in data. However, its effectiveness depends on careful selection of bandwidth, weighting functions, and variables [32].

This method has been widely used in epidemiological studies of infectious diseases to investigate the spatial determinants of a given disease [33,34,35]. This analysis was performed for vaccination coverage separately for females, males, and both, and subsequently the model with the best performance was evaluated by considering the highest R^2^ and Akaike information criterion (AIC). In addition, bandwidth was configured to adapt to the GWR, following the Gauss distribution on the geographical weighting function, and the default method for drop-1 cross-validation. Four independent variables entered the regression analysis: municipal performance, educational performance, family health strategy coverage, and primary care coverage.

To detect possible multicollinearity problems, variance inflation factor (VIF) analysis was applied, considering values lower than 5 to rule out the hypothesis of multicollinearity [36]. These analyses were performed in the R software (Version 4.4) using the GWmodel, car, and spdep packages.

The results were plotted on choropleth maps using QGIS software (version 3.4).

### 2.5. Ethics Committee

This study uses publicly accessible data, and such data are based on the Guidelines and Regulatory Standards for Research Involving Human Beings according to Resolution 530/2016 of the National Health Council.

## 3. Results

Between 2015 and 2022, 1,321,525 first doses were administered to children and adolescents between 9 and 14 years old in the state of Paraná (Table 2). Of these, 727,529 (55.05%) doses were administered to girls and 593,996 (44.95%) to boys. The highest number of doses administered to girls (62,502) was observed in 2015 for 9-year-olds. For boys, the highest number of doses administered was in 2018 (59,842 doses for boys aged 11 years).

Table 3 shows the number of second doses of the vaccine. A total of 1,030,182 doses were administered between 2015 and 2022, a reduction of 291,343 doses (22.04%). Among the total, 631,482 (61.30%) were administered to girls and 398,700 (38.70%) to boys.

**Figure 2:**
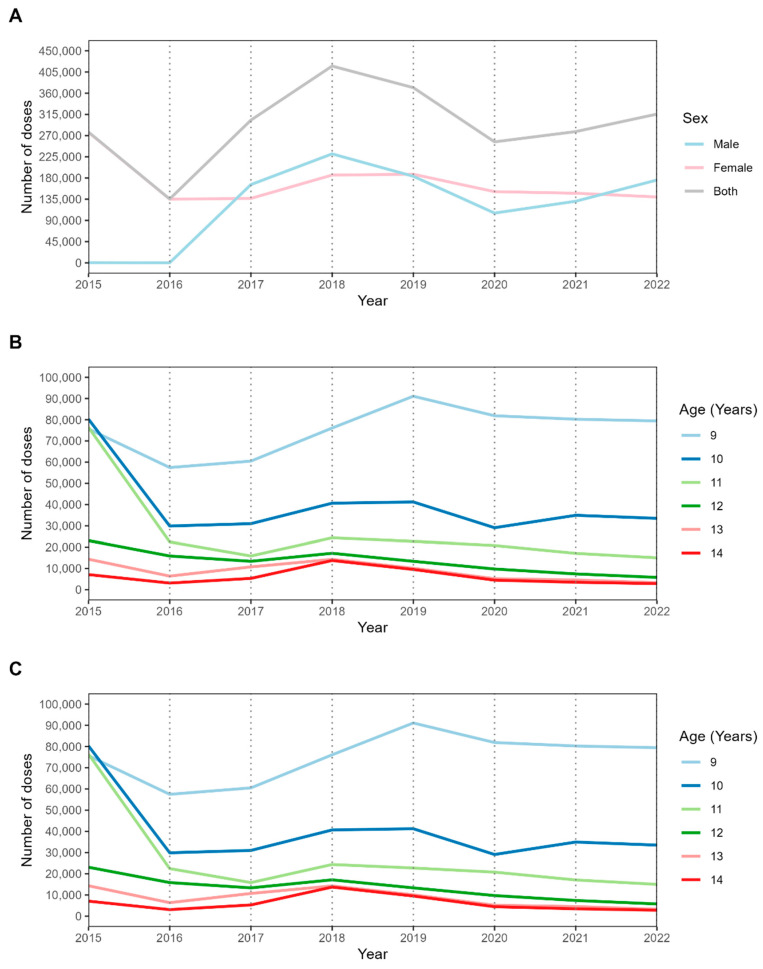
Temporal evolution of the number of doses administered in Paraná between 2015 and 2022. In (**A**) by sex (Male, Female, and Both sexes), (**B**) by age group (9 to 14 years) for females, and (C) by age group (9 to 14 years) for males.

Figure 2 shows the administration of all doses of the HPV vaccine for boys and girls. Panel A shows that the peak of immunizations occurred in 2018, followed by a decline until 2020. Thereafter, there was a positive trend until 2022. Panel B shows the distribution of vaccines for girls. Girls aged 9 were the most vaccinated. Boys, as illustrated in panel C, in the 11-year-old group received the largest number of doses. For both sexes, a decline in vaccination was observed in mid-2020.

**Figure 3:**
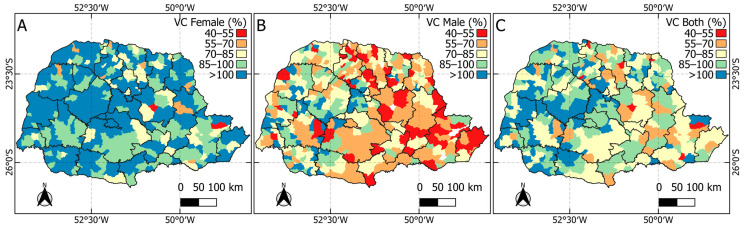
Distribution of HPV vaccination coverage for the female population (**A**), male population (**B**), and both (**C**).

Figure 3 shows HPV vaccination coverage in municipalities in the state of Paraná between 2015 and 2022. There is heterogeneity in coverage between girls (panel A) and boys (panel B). Vaccination coverage with the first dose of the vaccine reached 95.17% for the female population, 64.67% for the male population, and 79.57% when both sexes are considered. In addition, it was observed that 202 municipalities (50.62%) had coverage above 90% when considering both sexes. When analyzing only the female sex, 321 municipalities (80.45%) reached the recommended coverage, while for the male sex, only 95 municipalities (23.80%) reached this level.

Regarding spatial analysis, Moran’s I indices for vaccination coverage were calculated separately for girls, boys, and for both sexes combined. The values obtained were 0.17 for girls, 0.23 for boys, and 0.22 for both sexes, with a *p*-value < 0.05 in all cases. These results indicate a significant spatial dependence in the distribution of vaccination coverage rates within these populations, suggesting that vaccination rates are not randomly distributed, but exhibit distinct spatial patterns that may be associated with local or regional factors. Table 4 presents the results of the geographically weighted regression. It can be observed that the model presented the best performance parameters when analyzing vaccination coverage for both sexes due to the higher R^2^ and lower AIC.

**Figure 4:**
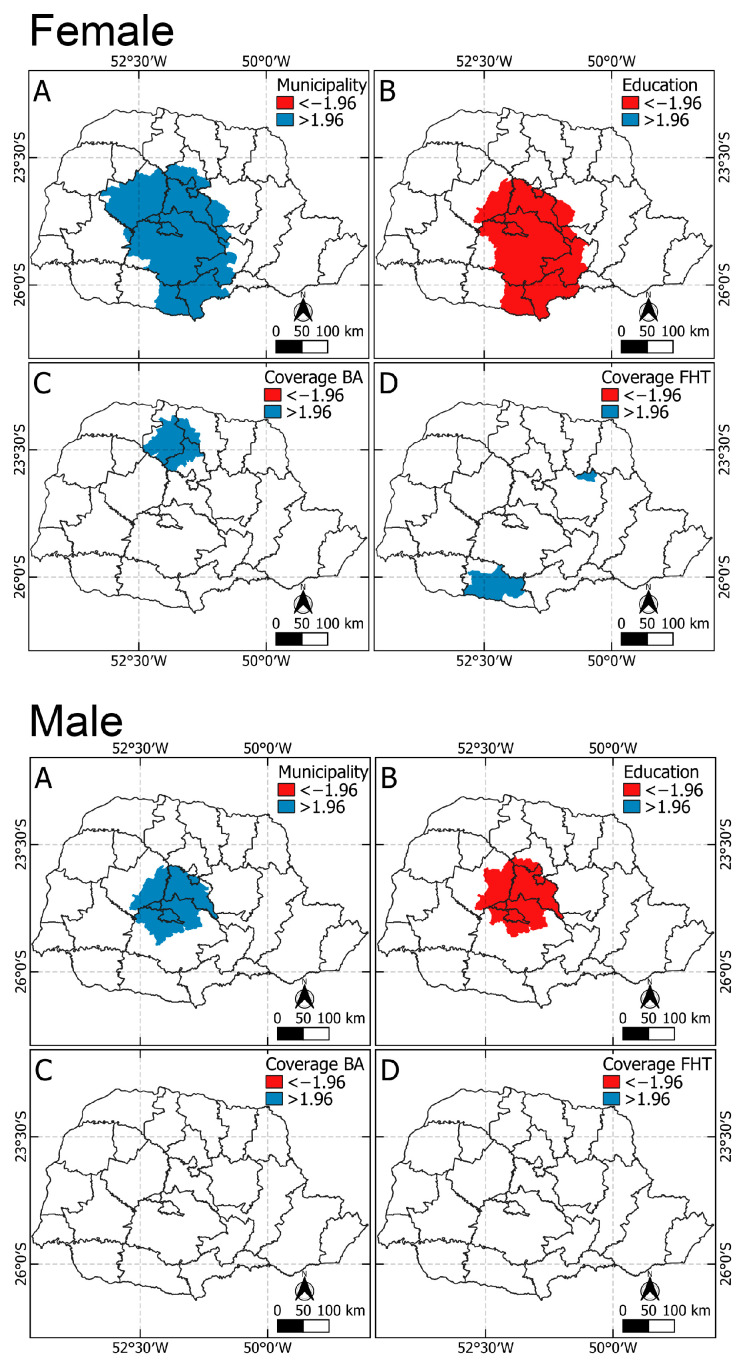

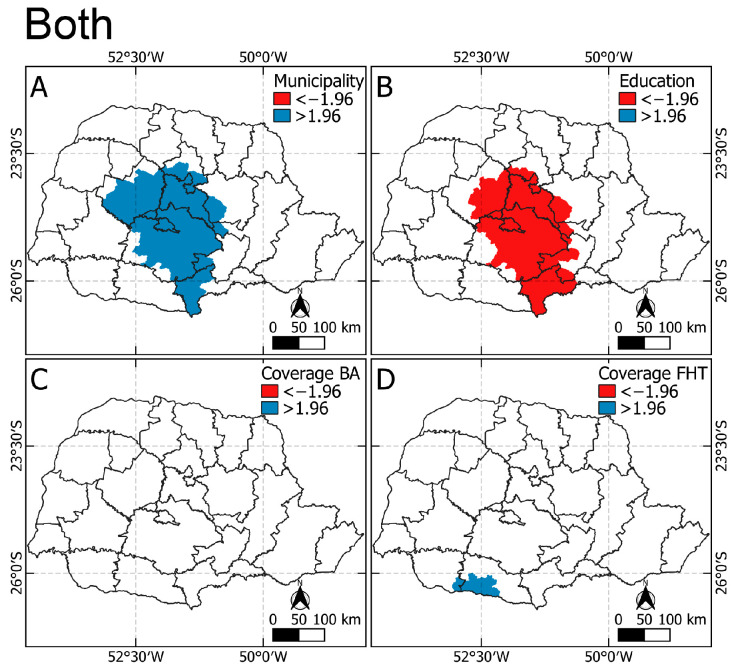
Significant results from geographically weighted regression, Brazil, 2024. Spatial distribution of municipalities with significant associations between independent variables and vaccination coverage by sex (female, male, and both): direct relationships are indicated in blue (t > 1.96), and inverse relationships in red (t < −1.96).

Figure 4 shows the municipalities with significant results (t < −1.96 or t > 1.96). For the interpretation of this analysis, the values in blue represent t values > 1.96, which means that there was a significant and direct relationship, i.e., the higher the values of the independent variables, the higher the value of the outcome variable. The red colors represent t values < −1.96 and presented a significant inverse relationship, meaning the lower the value of the independent variable, the higher the value of the outcome variable. In this sense, the municipal performance variable was significant (t > 1.96) for increasing vaccination coverage for girls, boys, and both sexes who were closer to regions 4, 5, 6, 7, 11, and 15. The family strategy coverage variable was significant for girls and both sexes surrounding regions 7, 8, and 21. The education variable was inversely significant for girls, boys and both sexes in cities closer to regions 4, 5, 6, 11, 21, and 22. Primary care coverage was not significant for boys or both sexes.

## 4. Discussion

To the best of our knowledge, this is one of the first studies to evaluate HPV vaccination coverage and its relationship to sociodemographic variables in the state of Paraná, Brazil. Our findings suggest that municipal performance, educational performance, family health strategy coverage, and primary care coverage, as identified through spatial analysis, were key factors influencing the outcomes.

An important piece of information found in the study was related to the coverage of each of the two application doses. The data showed that there was a reduction between the first and second vaccine doses, indicating the existence an instability in the program in not completing the two-dose vaccination schedule. The vaccine has a significant impact on the annual 13% reduction in HPV among girls and boys who are fully vaccinated [37].

In the state of Paraná, there was a tendency for a gradual reduction over time in the immunization rate across all age groups and for both sexes. Despite presenting comparatively higher socioeconomic indicators than other regions (HDI, per capita income, and access to basic services such as education and health) Paraná also faces the challenges of social inequalities, especially in urban areas [38]. Some states in Brazil, such as Pará, Tocantins, and Goiás, among others, had difficulty achieving vaccine coverage [20]. One explanation for the lack of coverage may be associated with the way in which the information was spread and families’ lack of understanding about the vaccine’s effectiveness and contraindications. The concerns of both health-care professionals and the general population when the vaccine was introduced were underestimated [39].

Another significant finding was the disparity in vaccination coverage between boys and girls. Coverage for females exceeded the recommended levels, while coverage for males fell short. Vaccinating early adolescents (ages 9–14) is indispensable because it is a cost-effective and efficient strategy for preventing cervical cancer, especially in resource-limited settings. Additionally, vaccinating males early is effective in preventing anogenital warts, as well as penile and anorectal cancers [38].

In 2014, the Ministry of Health approved the implementation of free HPV vaccination in the National Immunization Program (NIP) schedule for girls aged 9 to 13. This age group would benefit most due to their high antibody production and lower exposure to the virus [40]. Later, in 2017, the program was expanded to include girls aged 9 to 14 and introduced to the male population aged 11 to 14 or 9 to 26 living with HIV/AIDS, as well as transplant recipients and cancer patients [41]. The female population might have higher vaccination coverage, given it was included earlier in the NIP. This was observed in our study, where approximately 69.4% (1,293,255) of the total doses of vaccines were administered to this population. Boys were not a priority in the WHO vaccination strategy, as their contribution to female protection is limited and only relevant when coverage for girls reaches the recommended level [39]. However, this protection reaches heterosexual men and women, which is why countries such as Brazil have introduced vaccination for boys with the aim of reducing viral load, infections, and inequality in public health [41]. Despite this discrepancy between the sexes, the state of Paraná achieved greater coverage than the rest of the country for both sexes. In Brazil, vaccination coverage with the first dose reached 76% for girls and 42% for boys. For the second dose, girls had slightly higher coverage than the rest of Brazil, though not reaching 60%. Boys, on the other hand, had over 10% higher coverage compared to the rest of the country (27%) [42].

All in all, regarding the impact of vaccination programs, population level, and herd immunity, there was a 64% decrease in the overall prevalence of HPV types 16 and 18 in the post-vaccination period [43]. According to those authors, countries with low coverage did not show a decrease in the disease. These data reinforce the importance of completing the vaccination schedule for its effectiveness and efficiency in reducing HPV prevalence.

Considering the spatial distribution of vaccination coverage rates in the state of Paraná, half of the municipalities achieved 90% coverage for both sexes. However, there is a large difference between girls (80% of municipalities achieved the recommended coverage) and boys (only 23% of municipalities achieved coverage).

There were positive associations between vaccination coverage, socioeconomic conditions, and income in the state as a whole. The higher the socioeconomic level of the population, the better the health indicators. Studies conducted in the United States have shown that the higher the income and level of education and the greater the knowledge, the greater the use of the vaccine [42]. Regarding the variable of educational performance, there was an inverse correlation, that is, the lower the educational performance, the greater the vaccination coverage. This might have occurred due to the fact that 51% of the municipalities have fewer than 10,000 inhabitants [38] and little educational structure, such as only elementary school, resulting in the population seeking health services in larger municipalities with better structure.

The Brazilian health system is based on universality, longitudinality, horizontality, equity, and comprehensiveness for every individual in the national territory. Vaccines are distributed free of charge, and access to vaccination programs is meant to be equal for everyone. Nevertheless, difficulty presents itself not only in access but also in understanding the importance of the vaccine. A study has explored the relationship between HPV vaccination and sociodemographic variables. For instance, research conducted in Norway found that parental education and income levels were significantly associated with vaccination rates [43], revealing socioeconomic disparities similar to those observed in this study.

Another relevant finding regarding vaccination coverage is the performance of primary care and the family health strategy. Primary care did not present statistical significance; however, the family health strategy was significant in two regions (southwest and general fields). Despite the progress in health care, there are still inequalities in the provision of services for vulnerable populations based on social stratification [44], which can be evidenced by the low coverage of the HPV vaccine. Efforts by the family health strategy to qualify primary care coverage are important to reduce access barriers and contribute to the formulation of new health policies [45].

This study highlighted the need for action by the primary health-care system and the family health strategy to increase vaccination coverage. Primary health care and the family health strategy are responsible for promoting, preventing, providing qualified care, and integrating practices for the entire population. Despite this, there is still a need to expand and consolidate a strong primary health-care system that organizes health-care networks, integrates itself efficiently with surveillance systems, and stimulates the pursuit of professional qualifications, while also expanding its coverage and access to services so that it finally reduces inequalities [46].

As for the spatial analysis presented in this study, it is an important approach in the context of HPV vaccination in Brazil. While some international studies have explored geographic disparities in vaccine coverage, few have applied a detailed spatial analysis at the municipal level, particularly in regions with significant socioeconomic variation like Paraná. This spatial dimension allows for a more nuanced understanding of local disparities, offering insights into how health policies and vaccination programs can be tailored to address regional needs and improve overall coverage.

A key recommendation for future efforts is to reduce the vaccination coverage gap between boys and girls and to achieve a higher percentage of the population vaccinated. Future campaigns should focus on specific strategies using social marketing techniques. Countries like Australia, Mexico, and Peru have effectively utilized social marketing strategies in their vaccination campaigns to more successfully reach and engage their target populations [18]. Thus, efforts to raise awareness among boys need to be strengthened, with campaigns targeting families and communities to educate them on the importance of vaccinating boys, emphasizing the prevention of HPV-related cancers. Moreover, leveraging the FHS is important, as municipalities with well-established FHS infrastructure showed better vaccination rates. This suggests that strengthening primary care teams could improve coverage, particularly in underperforming areas. Educational barriers should also be addressed, with public health campaigns highlighting the importance of vaccination, especially in regions with lower educational levels. Schools can play a key role in increasing awareness and facilitating access to vaccines. Finally, improving data collection and tracking is essential, ensuring that municipalities have robust monitoring systems to better capture population movements, which can distort vaccination statistics. By implementing these strategies and addressing administrative and logistical challenges, Paraná can improve its HPV vaccination rates and achieve more equitable coverage across all populations.

One limitation of this study was the use of secondary data from the Health Information System database, which may occasionally be incomplete. Despite that, the data are official and a tool that has the advantage of presenting low cost, ease of monitoring, and broad population coverage. Another limitation was the difference in the timing of vaccine availability for girls and boys, which generated a discrepancy in data volume, since the program began at different times for each gender.

## 5. Conclusions

This study showed suboptimal HPV vaccination coverage in most years and in both sexes, although in some regions, the coverage rate for girls reached the recommended level. This situation is troublesome, since the vaccine brings future benefits. The factors analyzed influenced the low adherence and vaccination coverage. Given that half of the municipalities in the state achieved 90% coverage for both sexes, it is essential that whichever strategy is used in these municipalities be disseminated and adopted by the others. New studies are vital to investigate the barriers to the adherence of this immunobiological, since reaching the coverage target will bring significant advances to public health by reducing cervical cancer mortality rates. Analysis studies using spatial statistics are necessary to strengthen and unravel the disparities that exist between the regions of the state of Paraná.

## Figures and Tables

**Table 1:** Data source for the variables used in this study.

Source	Variable	Reference
Department of Informatics of the Unified Health System (DATASUS)	Population estimate of females and males aged 9 to 14 years old Doses of the quadrivalent HPV vaccine	[24]
Paraná Institute for Economic and Social Development (IPARDES)	Municipal performance Educational performance Family health strategy coverage Primary care coverage	[27]
Brazilian Institute of Geography and Statistics (IBGE)	Shapefile of continuous cartographic bases	[22]

**Table 2:** Total number of first doses of the quadrivalent HPV vaccine administered to people aged 9 to 14 in Paraná between 2015 and 2022.

Year	Female						Male					
	Age											
	9	10	11	12	13	14	9	10	11	12	13	14
2015	62,502	52,818	38,542	6434	3506	415	79	86	65	22	33	31
2016	40,873	13,003	8387	5642	2157	192	19	22	31	28	27	18
2017	40,464	11,964	6625	5889	4580	2046	951	1308	27,409	51,303	37,193	12,995
2018	55,981	19,766	11,493	7970	6704	6300	1096	1699	59,842	34,670	23,789	19,573
2019	60,136	10,592	6768	3610	3048	2829	955	2674	55,049	10,079	8004	6602
2020	55,992	6814	9873	2487	1257	1091	771	799	44,304	5195	1691	1398
2021	55,699	9359	6794	2128	1236	841	540	465	55,482	8921	2613	1091
2022	55,451	8601	5180	1675	996	819	25,699	24,130	53,515	7459	2917	1354
Total	427,098	132,917	93,662	35,835	23,484	14,533	30,110	31,183	295,697	117,677	76,267	43,062

**Table 3:** Total number of second doses of the quadrivalent HPV vaccine administered to people aged 9 to 14 in Paraná between 2015 and 2022.

Year	Female						Male					
	Age											
	9	10	11	12	13	14	9	10	11	12	13	14
2015	13,120	27,385	37,785	16,552	10,767	6598	27	59	42	24	14	33
2016	16,557	16,846	14,012	10,167	4169	2870	5	23	21	28	29	16
2017	20,010	18,985	9167	7385	6096	3192	140	193	954	9336	15,143	9069
2018	20,071	20,794	12,723	8978	7414	7227	321	718	15,488	29,202	23,644	20,805
2019	30,916	30,601	15,900	9704	6983	6629	410	1089	26,591	32,227	22,103	17,770
2020	25,833	22,245	10,870	7225	3886	3352	216	600	19,570	18,888	6520	5531
2021	24,511	25,555	10,247	5268	3304	2622	256	412	21,479	25,331	9062	4987
2022	23,958	24,884	9778	4092	2254	1995	247	413	21,507	25,266	8735	4156
Total	174,976	187,295	120,482	69,371	44,873	34,485	1622	3507	105,652	140,302	85,250	62,367

**Table 4:** Geographically weighted regression results, Brazil, 2024.

Variable	Min	1st Q	Median	3rd Q	Max
Female					
Intercept	55.00	67.44	85.51	96.01	131.13
Municipal performance	−27.71	29.13	45.73	77.83	143.88
Education	−75.68	−42.12	−31.32	−6.16	51.37
PC Coverage	−0.21	−0.09	0.07	0.29	0.50
FHS Coverage	−0.31	−0.19	−0.004	0.16	0.32
R^2^	0.19				
AIC	3580.23				
Male					
Intercept	46.19	67.92	76.62	86.58	121.52
Municipal performance	−37.32	6.89	26.90	56.15	100.85
Education	−79.11	−35.74	−29.22	−16.90	32.02
PC Coverage	−0.12	−0.06	0.00	0.04	0.12
FHS Coverage	−0.13	−0.06	0.00	0.05	0.23
R^2^	0.22				
AIC	3505.45				
Both					
Intercept	50.65	72.05	80.73	89.29	118.57
Municipal performance	−41.73	15.59	30.34	68.24	123.38
Education	−77.51	−39.76	−28.64	−10.74	27.57
PC Coverage	−0.17	−0.07	0.05	0.15	0.30
FHS Coverage	−0.21	−0.11	0.00	0.12	0.25
R^2^	0.23				
AIC	3464.58				

## Data Availability

The data are publicly available in an open repository and can be accessed through the following link: https://doi.org/10.6084/m9.figshare.26838736 (accessed on 25 September 2024).

## References

[1] World Health Organization Cervical Cancer. https://www.who.int/news-room/fact-sheets/detail/cervical-cancer#:~:text=Key%20facts.

[2] Women’s Health Month: Cervical Cancer Awareness. https://www.piedmont.org/living-real-change/womens-health-month-cervical-cancer-awareness.

[3] Cancer Research UK Cervical Cancer Statistics. https://www.cancerresearchuk.org/health-professional/cancer-statistics/statistics-by-cancer-type/cervical-cancer#heading-Zero.

[4] PAHO HPV e Câncer do Colo do Útero. https://www.paho.org/en/topics/cervical-cancer.

[5] Instituto Nacional de Câncer (Brasil) (2022). Estimativa 2023: Incidência de Câncer no Brasil.

[6] World Health Organization Cervical Cancer Fact Sheet. https://www.who.int/es/news-room/fact-sheets/detail/cervical-cancer.

[7] Pennella R.A., Ayers K.A., Brandt H.M. (2020). Understanding How Adolescents Think about the HPV Vaccine. Vaccines.

[8] Senkomago V., Henley S.J., Thomas C.C., Mix J.M., Markowitz L.E., Saraiya M. (2019). Cânceres atribuíveis ao papilomavírus humano—Estados Unidos, 2012–2016. MMWR Morb. Mortal. Wkly. Rep..

[9] Castle P.E., Silva V.R.S., Consolaro M.E.L., Kienen N., Bittencourt L., Pelloso S.M., Partridge E.E., Pierz A., Dartibale C.B., Uchimura N.S. (2019). Participation in Cervical Screening by Self-collection, Pap, or a Choice of Either in Brazil. Cancer Prev. Res..

[10] Shin M.B., Liu G., Mugo N., Garcia P.J., Rao D.W., Bayer C.J., Barnabas R.V. (2021). A Framework for Cervical Cancer Elimination in Low-and-Middle-Income Countries: A Scoping Review and Roadmap for Interventions and Research Priorities. Front. Public Health.

[11] Federação Brasileira das Associações de Ginecologia e Obstetrícia (Febrasgo) Diagnóstico de Câncer de Mama e Colo de Útero Caem 23.4% durante a Pandemia. https://www.febrasgo.org.br/pt/noticias/item/1242-diagnostico-de-cancer-de-mama-e-colo-de-utero-caem-23-4-durante-a-pandemia.

[12] dos Santos L., Roszkowski I., Pujals C., de Oliveira R., Pelloso F., Pelloso Borghesan D., Romani I., Romanio Bitencourt M., Jacinto Alarcão A., Dias Marques V. (2023). Análise comparativa da mortalidade por câncer de mama e realização de mamografia nas unidades federativas do Brasil-2015 a 2021. Asian Pac. J. Cancer Prev..

[13] Fundação Oswaldo Cruz (FIOCRUZ) Imunização, Uma Descoberta da Ciência Que Vem Salvando Vidas Desde o Século XVIII. https://butantan.gov.br/noticias/imunizacao-uma-descoberta-da-ciencia-que-vem-salvando-vidas-desde-o-seculo-xviii.

[14] Bergman H., Buckley B.S., Villanueva G., Petkovic J., Garritty C., Lutje V., Riveros-Balta A.X., Low N., Henschke N. (2019). Comparison of different human papillomavirus (HPV) vaccine types and dose schedules for prevention of HPV-related disease in females and males. Cochrane Database Syst. Rev..

[15] Falcaro M., Castañon A., Ndlela B., Checchi M., Soldan K., Lopez-Bernal J., Elliss-Brookes L., Sasieni P. (2021). The effects of the national HPV vaccination programme in England, UK, on cervical cancer and grade 3 cervical intraepithelial neoplasia incidence: A register-based observational study. Lancet.

[16] Lei J., Ploner A., Elfström K.M., Wang J., Roth A., Fang F., Sundström K., Dillner J., Sparén P. (2020). HPV Vaccination and the Risk of Invasive Cervical Cancer. N. Engl. J. Med..

[17] Palmer T., Wallace L., Pollock K.G., Cuschieri K., Robertson C., Kavanagh K., Cruickshank M. (2019). Prevalence of cervical disease at age 20 after immunisation with bivalent HPV vaccine at age 12–13 in Scotland: Retrospective population study. BMJ.

[18] Santos W.M., Santos D.M., Fernandes M.S. (2023). HPV immunization in Brazil and proposals to increase adherence to vaccination campaigns. Rev. De Saúde Pública.

[19] PAHO Despite Vaccination Gains, 1.2 Million Children under One Remain Unprotected in the Americas. https://www.paho.org/en/news/18-4-2024-despite-vaccination-gains-12-million-children-under-one-remain-unprotected-americas.

[20] Moura L.d.L., Codeço C.T., Luz P.M. (2021). Human papillomavirus (HPV) vaccination coverage in Brazil: Spatial and age cohort heterogeneity. Rev. Bras. De Epidemiol..

[21] STROBE (2023). Checklists–STROBE. STROBE. https://www.strobe-statement.org/checklists/.

[22] IBGE Instituto Brasileiro de Geografia e Estatística. https://www.ibge.gov.br/.

[23] Uchimura L.Y.T., Felisberto E., Fusaro E.R., Ferreira M.P., Viana A.L.D.Á. (2017). Evaluation performance in health regions in Brazil. Rev. Bras. Saúde Matern. Infant..

[24] DATASUS. https://datasus.saude.gov.br/.

[25] Brasil Ministério da Saúde. Nota Técnica no 41/2024-CGICI/DPNI/SVSA/MS. https://www.gov.br/saude/pt-br/centrais-de-conteudo/publicacoes/notas-tecnicas/2024/nota-tecnica-no-41-2024-cgici-dpni-svsa-ms/view.

[26] PAHO (2019). Metodologia Para o Cálculo de Cobertura da Vacina Contra o HPV na Região das Américas.

[27] IPARDES (2018). Índice IPARDES de Desempenho Municipal. https://www.ipardes.pr.gov.br/sites/ipardes/arquivos_restritos/files/documento/2019-09/Metodologia%20IPDM%202016.pdf.

[28] Brasil Nota Técnica–Relatório de Cobertura da Atenção Básica. https://egestorab.saude.gov.br/paginas/acessoPublico/relatorios/nota_tecnica/nota_tecnica_relatorio_de_cobertura_AB.pdf.

[29] World Health Organization (2024). Cobertura de Imunização. https://www.who.int/news-room/fact-sheets/detail/immunization-coverage#:~:text=In%202020%2C%20the%20World%20Health,target%20of%20reaching%2090%25%20coverage.

[30] Moran P.A.P. (1950). Notes on Continuous Stochastic Phenomena. Biometrika.

[31] Li H., Calder C., Cressie N.A. (2007). Beyond Moran’s I: Testing for spatial dependence based on the spatial autoregressive model. Geogr. Anal..

[32] Kiani B., Sartorius B., Lau C.L., Bergquist R. (2024). Mastering geographically weighted regression: Key considerations for building a robust model. Geospat. Health.

[33] Mayfield H.J., Lowry J.H., Watson C.H., Kama M., Nilles E.J., Lau C.L. (2018). Use of geographically weighted logistic regression to quantify spatial variation in the environmental and sociodemographic drivers of leptospirosis in Fiji: A modelling study. Lancet Planet. Health.

[34] Bui L.V., Mor Z., Chemtob D., Ha S.T., Levine H. (2018). Use of Geographically Weighted Poisson Regression to examine the effect of distance on Tuberculosis incidence: A case study in Nam Dinh, Vietnam. PLoS ONE.

[35] Cheng E.M., Atkinson P.M., Shahani A.K. (2011). Elucidating the spatially varying relation between cervical cancer and socio-economic conditions in England. Int. J. Health Geogr..

[36] Kim J.H. (2019). Multicollinearity and misleading statistical results. Korean J. Anesthesiol..

[37] Hoes J., Woestenberg P.J., Bogaards J.A., King A.J., De Melker H.E., Berkhof J., Hoebe C.J., Van Der Sande M.A., Van Benthem B.H. (2021). Population Impact of Girls-Only Human Papillomavirus 16/18 Vaccination in The Netherlands: Cross-Protective and Second-Order Herd Effects. Clin. Infect. Dis..

[38] Shin H., Jeon S., Cho I., Park H. (2022). Factors Affecting Human Papillomavirus Vaccination in Men: Systematic Review. JMIR Public Health Surveill..

[39] Bloem P. (2020). Estratégia de vacinação contra HPV. Rev. Imunizações SBIm.

[40] Instituto Nacional de Câncer José Alencar Gomes da Silva (INCA) (2021). Detecção Precoce do Câncer.

[41] Villa L.L. (2020). Vacina papilomavírus humano (HPV): Atualização e perspectivas. Rev. Imunizações SBIm.

[42] Drolet M., Bénard É., Boily M.-C., Ali H., Baandrup L., Bauer H., Beddows S., Brisson J., Brotherton J.M.L., Cummings T. (2015). Population-level impact and herd effects following human papillomavirus vaccination programmes: A systematic review and meta-analysis. Lancet Infect. Dis..

[43] Feiring B., Laake I., Molden T., Cappelen I., Håberg S.E., Magnus P., Steingrímsdóttir Ó.A., Strand B.H., Stålcrantz J., Trogstad L. (2015). Do parental education and income matter? A nationwide register-based study on HPV vaccine uptake in the school-based immunisation programme in Norway. BMJ Open.

[44] Fernández-Feito A., Lana A., Parás Bravo P., Pellico López A., Paz-Zulueta M. (2020). Knowledge of the Human Papillomavirus by Social Stratification Factors. Nurs. Res..

[45] Giovanella L., Bousquat A., Schenkman S., de Almeida P.F., Sardinha L.M.V., Vieira M.L.F.P. (2021). Cobertura da Estratégia Saúde da Família no Brasil: O que nos mostram as Pesquisas Nacionais de Saúde 2013 e 2019. Ciênc. Saúde Colet..

[46] PAHO Relatório 30 anos de SUS, que SUS para 2030? Brasília, DF, 2018. https://iris.paho.org/handle/10665.2/49663.

